# Evaluating beluga (*Delphinapterus leucas*) blow samples as a potential diagnostic for immune function gene expression within the respiratory system

**DOI:** 10.1093/conphys/coac045

**Published:** 2022-07-03

**Authors:** Justin T Richard, Krystle Schultz, Caroline E C Goertz, Roderick C Hobbs, Tracy A Romano, Becky L Sartini

**Affiliations:** Department of Fisheries, Animal and Veterinary Science, University of Rhode Island, 9 E Alumni Drive, Kingston, RI 02881, USA; Mystic Aquarium, a Division of Sea Research Foundation, 55 Coogan Blvd, Mystic, CT 06355, USA; Department of Fisheries, Animal and Veterinary Science, University of Rhode Island, 9 E Alumni Drive, Kingston, RI 02881, USA; Alaska SeaLife Center, 301 Railway Avenue, Seward, AK 99664, USA; Retired from Marine Mammal Laboratory, Alaska Fisheries Science Center, 7600 Sand Point Way NE, F/AKC3, Seattle, WA 98115-6349, USA; Mystic Aquarium, a Division of Sea Research Foundation, 55 Coogan Blvd, Mystic, CT 06355, USA; Department of Fisheries, Animal and Veterinary Science, University of Rhode Island, 9 E Alumni Drive, Kingston, RI 02881, USA

## Abstract

Evaluating respiratory health is important in the management of cetaceans, which are vulnerable to respiratory diseases. Quantifying the expression of genes related to immune function within the respiratory tract could be a valuable tool for directly assessing respiratory health. Blow (exhale) samples allow DNA analysis, and we hypothesized that RNA could also be isolated from blow samples for gene expression studies of immune function. We evaluated the potential to extract RNA from beluga blow samples and tested whether transcripts associated with immune function could be detected with endpoint polymerase chain reaction. A total of 54 blow samples were collected from clinically healthy aquarium belugas (*n* = 3), and 15 were collected from wild belugas temporarily restrained for health assessment in Bristol Bay, Alaska (*n* = 9). Although RNA yield varied widely (range, 0–265.2 ng; mean = 85.8; SD = 71.3), measurable RNA was extracted from 97% of the samples. Extracted RNA was assessed in 1–6 PCR reactions targeting housekeeping genes (*Rpl8*, *Gapdh* or *ActB*) or genes associated with immune function (*TNF*α, *IL-12p40* or *Cox-2*). Fifty of the aquarium samples (93%) amplified at least one transcript; overall PCR success for housekeeping genes (96/110, 87%) and genes associated with immune function (90/104, 87%) were similarly high. Both RNA yield and overall PCR success (27%) were lower for wild beluga samples, which is most likely due to the reduced forcefulness of the exhale when compared with trained or free-swimming belugas. Overall, the high detection rate with PCR suggests measuring gene expression in blow samples could provide diagnostic information about immune responses within the respiratory tract. While further study is required to determine if quantitative gene expression data from blow samples is associated with disease states, the non-invasive nature of this approach may prove valuable for belugas, which face increasing anthropogenic disturbances.

## Teaser

We describe a method for the isolation of total RNA from blow (exhale) samples collected from beluga whales. Transcripts used to assess immune function in cetaceans were amplified using these samples, establishing the potential to quantify gene expression in blow samples as a minimally invasive diagnostic of respiratory health.

## Introduction

Many cetacean populations are exposed to significant levels of environmental contaminants and other anthropogenic stressors, as well as habitat changes resulting from climate change and increased human activities (Hobbs *et al.,* 2019). These stressors could suppress the immune system, making individuals more susceptible to disease, as has been documented for a variety of contaminants (Desforges *et al.,* 2016). Therefore, the study of cetacean immunology is an important component of cetacean conservation efforts. Several approaches are available to assess cetacean immune function (reviewed in Beineke *et al.,* 2010), including the quantification of cytokine gene expression. As cytokines mediate the immune system, levels of cytokine gene expression are an indicator of leukocyte function and thus provide a diagnostic indicator of cetacean health (Beineke *et al.,* 2007; Sitt *et al.,* 2008). This approach has been used in both free-ranging and managed-care cetaceans (Sitt *et al.,* 2016; Fair *et al.,* 2017). An advantage of this approach is that it does not require blood sampling and is readily applied to live, free-swimming animals via biopsy sampling (Buckman *et al.,* 2011). Additionally, tissue-specific or systemic immune responses can be investigated depending on the tissue sampled.

For cetacean health assessments, the respiratory system is of special interest. Due to their surfacing and breath-hold behaviour, cetaceans may be particularly vulnerable to respiratory diseases (Venn-Watson *et al.,* 2015; Raverty *et al.,* 2017). Exposure to oil spills has been associated with respiratory disease, which can impair reproduction or lead to mortality (Lane *et al.,* 2015; Venn-Watson *et al.,* 2015; Pasamontes *et al.,* 2017). The ability to directly assess immune function within the respiratory system by measuring cytokine gene expression would improve our ability to monitor cetaceans and assess the impact of anthropogenic disturbances, especially if the tissue sample could be collected using non-invasive methods.

Hunt *et al.* (2013) posited that blow (exhale) sampling of cetaceans could provide a non-invasive source of RNA for transcriptome analysis, which may relate to respiratory health. Blow samples provide a source of DNA from the cetacean host (Frère *et al.,* 2010) and DNA or RNA from microbes associated with the respiratory tract (Acevedo-Whitehouse *et al.,* 2010; Groch *et al.,* 2020), demonstrating the utility of blow samples in molecular analyses. Leukocytes are a relatively common cytological finding in cetacean blow samples (Sweeney and Reddy, 2001), suggesting that transcripts related to immune function would be present in blow samples. A study of humpback whale (*Megaptera novaeangliae*) blow reported the detection of cetacean transcripts in pooled blow samples collected from 19 individuals (Geoghegan *et al.,* 2018), providing proof of concept. However, the potential for using a blow sample collected from an individual cetacean for immune function gene expression studies is unknown.

This study aimed to evaluate the use of blow samples collected from belugas in managed care and in the wild for immune function gene expression studies. Belugas that live in areas that are increasingly exposed to oil and gas development (Burek-Huntington *et al.,* 2015) would benefit from the development of additional respiratory health diagnostics. Immune function gene expression studies have been performed in this species using other tissue sources (Unal *et al.,* 2018). Additionally, the utility of blow sampling for DNA analysis (Richard *et al.,* 2017a) and the quantification of steroid hormones (Thompson *et al.,* 2014; Richard *et al.,* 2017b) have already been demonstrated in this species. In this study, a method for the isolation of total RNA from blow samples was described and the relationship between RNA yield and sample handling procedures was explored to determine the applicability of the sampling methods to field conditions. Isolated RNA was assessed in endpoint polymerase chain reactions for genes related to immune function as well as housekeeping genes commonly used in quantitative reverse transcription polymerase chain reaction studies in belugas and other cetaceans to determine if immune function genes are detectable in individual blow samples.

## Methods

### Study animals

Blow sampling of three adult, clinically healthy aquarium belugas [two males (DL1, DL3); one female (DL2)] was performed at Mystic Aquarium (Mystic, CT, USA) at irregular intervals between February 2015 and October 2016. The belugas were trained to position their head so that their blowhole was above the water’s surface, and then to exhale on cue. Blow samples were collected from nine wild belugas in Bristol Bay (BB), Alaska in May 2016 while they were being temporarily restrained for health assessment and the attachment of satellite transmitters (as described in Norman *et al.,* 2012). Wild beluga samples were collected under National Marine Fisheries Service Marine Mammal Research Permit #14245. This project was approved by the Institutional Animal Care and Use Committees of Mystic Aquarium (Project #12001) and the University of Rhode Island (Project #AN12-02-016).

### Blow sample collection and handling

Blow samples consisting of one to six successive exhales were collected into a sterile polypropylene 50-ml conical tube (Fisher Scientific, Waltham, MA, USA, #14-432-22) held inverted ~5 cm directly above the blowhole and tilted cranially by 30–45° as described in Richard *et al.* (2017a). To simulate sample collection from a free-swimming beluga, no attempt was made to clear environmental water from the blowhole prior to sample collection. BB samples consisted of three successive exhales. Aquarium samples consisted of a single exhale (*n* = 21) or three (*n* = 31), four (*n* = 1) or six (*n* = 1) successive exhales. The tubes were capped and placed on ice.

After collection, 1 ml of RNA*later*® (Ambion, Inc., Austin, TX, USA) was added to the tube within 15 min of collection. The tubes were rocked by hand to coat the inner surface of the tube with RNA*later*®. For BB samples, the tubes were held in coolers on ice packs for 4–6 h before being placed in a −20°C freezer, where they were stored for up to 7 days until shipment to the laboratory in a liquid nitrogen dry shipper. Aquarium samples were exposed to one of three handling regimes: transported to the laboratory on ice for immediate processing (60–90 min after collection), immediately frozen (within 20 min of collection) at −20°C until processing up to 2 weeks later, or placed in a cooler on ice packs for 6 h before being frozen at −20°C for up to 2 weeks (to simulate BB sampling) (Table 1).

**Table 1:** Sample sizes for aquarium (DL1, DL2 and DL3) and BB belugas with various handling regimes

Location	# of exhales	Handling regime	# of belugas	# of samples	# of samples per beluga
Aquarium	1	Fresh	3	10	DL1: 4 DL2: 3 DL3: 3
	1	Frozen	3	11	DL1: 5 DL2: 5 DL3: 1
	3	Fresh	2	10	DL1: 5 DL2: 5
	3	Frozen	3	11	DL1: 5 DL2: 5 DL3: 1
	3	Chilled/frozen	2	10	DL1: 5 DL2: 5
	4	Fresh	1	1	DL1: 1
	6	Fresh	1	1	DL2: 1
BB	3	Chilled/Frozen	9	15	3 belugas: 1 each 6 belugas: 2 each

Fresh: processed within 90 min of collection; frozen: frozen at −20°C for up to 2 weeks before processing; chilled/frozen: chilled on ice packs for 6 h, then frozen at −20°C for up to 2 weeks.

### RNA extraction and reverse transcription

Prior to RNA extraction, the tubes were thawed if necessary and the 50-ml conical tubes were again rolled by hand to coat the inner surface of the tubes with RNA*later*® and were then centrifuged for 10 min at 2060 × *g*. After pipetting up and down several times to dislodge material from the bottom of the tube, the fluid was pipetted from the conical tube into a 1.5-ml microcentrifuge tube. This tube was then centrifuged in a microcentrifuge for 10 min at 13400 × *g*.

The presence or absence of an observed cell pellet was then recorded. If a pellet was visible, the supernatant was removed completely before performing the RNA extraction protocol. If a pellet was not visible, all but ~20 μl of supernatant was removed by micropipette from the tube without disturbing the lower layer of liquid that presumably would contain cellular material. For BB samples, the presence of very fine sand in the samples made it difficult to determine if a cell pellet was present or not; thus ~20 μl of supernatant was left in all of the BB samples.

RNA was isolated using the Qiagen RNEasy Micro kit (Valencia, CA, USA). Following removal of RNA*later* supernatant, 150 μl of buffer RLT (with 0.01% β-mercaptoethanol) was added to the sample. Homogenization was performed by placing the tube in a Disruptor Genie® Cell Disruptor Homogenizer (Scientific Industries, Inc., Bohemia, NY, USA) for 2 min. The remainder of the extraction procedure followed the manufacturer’s ‘Fibrous Tissue’ protocol, which includes a Proteinase K protein digestion step. This protocol was utilized for these samples in an effort to remove any mucus that could restrict the flow through the spin column or contaminate the sample.

RNA concentration (ng/μl) and purity (A_260_/A_280_, the ratio of absorbance of 2 μl of sample at 260 and 280 nm) was assessed using a NanoDrop™ 8000 Spectrophotometer (Thermo Scientific, Waltham, MA, USA) according to the manufacturer’s instructions. The total yield was calculated assuming a 12-μl elution volume. The entire volume of extracted RNA was then immediately reverse-transcribed using the Qiagen QuantiTect® Reverse Transcription kit (Valencia, CA, USA) following the manufacturer’s protocol, including a no-template control and a no-reverse transcriptase control. The resulting cDNA was stored at −20°C.

### Primers

Primers used in this study were previously designed to be beluga-specific and to cross exon-exon boundaries (Sitt *et al.,* 2008; Noël *et al.,* 2014). Markers of immune function [interleukin-12 subunit p40 (*IL-12p40*), tumour necrosis factor alpha (*TNFα*) and cyclooxygenase-2 (*Cox-2*)] were amplified using primers designed by Sitt *et al.* (2008). Potential housekeeping genes [ribosomal protein L8 (*Rpl8*), glyceraldehyde-3-phosphate dehydrogenase (*Gapdh*) and cytoplasmic beta actin (*Actb*)] were amplified using primers designed by Noël *et al.* (2014). The number of PCR reactions attempted with each sample (range, 1–6) varied depending on template availability for both the sample and the reverse transcription (RT) negative controls associated with each sample; early trials utilized much more template in PCRs than was likely necessary in an effort to compensate for the unknown proportion of microorganism RNA present. Fifty aquarium samples and three BB samples were tested with more than one primer set. The three BB samples were tested with two primer sets (*Rpl8* and *TNF*α). For the aquarium samples, 3 samples were tested with two primer sets, 16 were tested with three primer sets, 1 sample was tested with four primer sets, 28 were tested with five primer sets and 2 were tested with all six primer sets.

### PCR methods

PCRs were carried out in 50 μl (1X reaction buffer, 1.5 mM MgCl_2_, 10 mM dNTPs, 2.5 μM forward and reverse primers and 2.5 U Taq polymerase) using 1–5 μl of cDNA template. PCR conditions described by Sitt *et al.* (2008) were used: 50°C for 2 min, 95°C for 15 min, 40 cycles of 94°C for 30 s, 55°C for 30 s, 72°C for 30 s and an extension step of 72°C for 10 min. For each reaction, a no-template PCR control as well as the two RT negative controls were also tested. An Eppendorf Mastercycler® EP (#5341) thermocycler was used for all PCRs.

The PCR product was loaded into a 2% agarose gel stained with GelRed™ (Phenix Research Products, Candler, NC, USA) for electrophoresis. Bands were visualized under UV light, and scoring was completed by visual examination. PCR performance was assessed through the presence or absence of the appropriate banding pattern.

### Sequencing

To ensure the amplification of the target RNA or to identify non-target bands that appeared upon electrophoresis, sequencing was performed on representative samples. For amplifications with a single band, the PCR product was purified using the Qiagen QIAquick® PCR Purification kit (Qiagen, Valencia, CA, USA). For reactions with multiple products, the bands were excised and cDNA was extracted using a Gel Extraction kit (Qiagen, Valencia, CA, USA). The cDNA was submitted for Sanger sequencing using an Applied Biosystems 3500XL Genetic Analyzer (Foster City, CA, USA) at the University of Rhode Island Genomics and Sequencing Center and resulting sequences were identified using the Basic Local Alignment Search Tool from the National Center for Biotechnology Information (http://blast.ncbi.nlm.nih.gov).

### Data presentation

Yields were expressed as the mean ± SD. Small sample sizes or samples clustered by individual that violated independence assumptions precluded rigorous statistical testing. The effects of variables of interest on RNA yield or PCR performance were shown using box plots created in *R* (R Core Team, 2020), where the box represents the interquartile range, the dark line represents the median and whiskers represent the minimum and maximum values (excluding outliers; >1.5× the interquartile range away from the minimum or maximum values). Each observation is plotted, with closed circles representing PCR success (target product was amplified) and open circles representing PCR failure (target product was not amplified).

## Results

### RNA extraction results

**Fig. 1:**
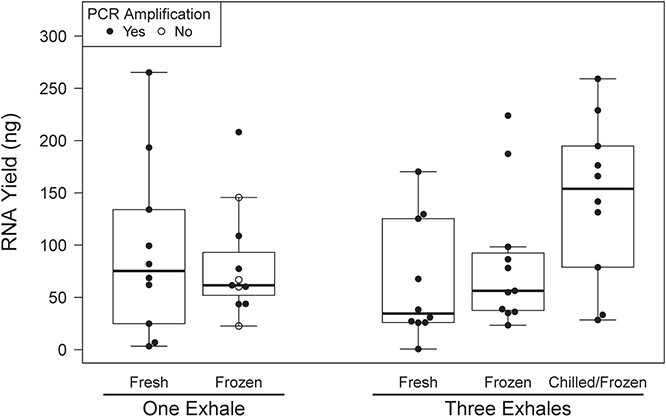
Total RNA yield and PCR success by number of exhales collected and sample handling protocol for aquarium beluga blow samples

**Fig. 2:**
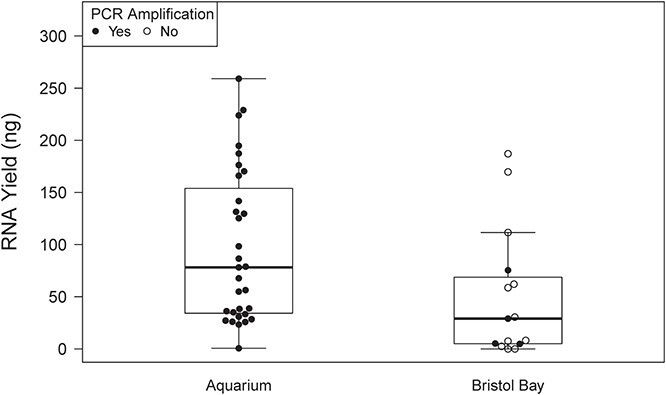
Total RNA yield and PCR success for three-exhale blow samples collected from aquarium and wild belugas in BB, Alaska

**Fig. 3:**
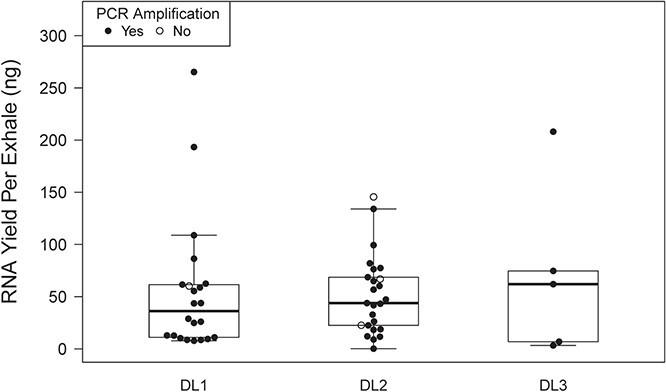
RNA yield per exhale and PCR success for blow samples collected from three individual belugas (DL1, DL2 and DL3).

Measurable RNA was extracted from all 54 of the aquarium samples and 13/15 of the BB samples. RNA yield varied greatly by sample (range, 0–265.2 ng; mean = 85.8; SD = 71.3) and by exhale (ng/exhale) (range, 0–265.2 ng; mean = 45.4; SD = 49.9). Results are summarized in Table 2. For aquarium samples, total yield was not proportional to the number of exhales collected (Fig. 1). Freezing the samples did not influence yield per exhale (not frozen: 57.7 ± 69.9 ng/exhale; chilled then frozen and frozen: 52.6 ± 42.4 ng/exhale). Chilling the three-exhale samples prior to freezing appeared to increase yield relative to other handling regimes, although all samples were within the range of other handling methods (Fig. 1). RNA yield per exhale was greater for aquarium samples (53.7 ± 52.7 ng) than for BB samples (15.7 ± 17.8 ng) (Fig. 2). Among aquarium samples, yield per exhale was similar across the three individuals sampled (Fig. 3). Presence of a cell pellet prior to extraction did not appear to influence yield; aquarium samples with pellets (17 samples) had a similar yield (42.9 ± 24.8 ng) to those without (37 samples, 58.7 ± 60.8 ng). The A_260_/A_280_ ratios varied widely by sample and were occasionally outside of the normal range for nucleic acid samples (range, −0.12–21.01). The 19 samples with yields >10 ng/μl had a A_260_/A_280_ of 1.57 ± 0.13.

**Table 2:** Results summary for RNA extraction from beluga blow samples

Sample source	# of exhales	# of samples	Total yield, ng (mean ± SD)	Total yield, ng (median)	Yield per exhale, ng (mean ± SD)	Yield per exhale, ng (median)	A_260_/A_280_ (mean ± SD)	% Samples with visible cell pellet	% Samples with PCR success
Aquarium	1	21	87.5 ± 66.4	66.9	87.5 ± 66.4	66.9	3.04 ± 4.08	10	81
	3	31	96.7 ± 72.1	78.1	32.3 ± 24.0	26.0	1.90 ± 1.04	42	100
BB	3	15	50.1 ± 59.7	29.0	15.7 ± 17.8	9.7	1.75 ± 1.21	NA	27

### PCR success and sequencing

Most samples provided template that was successfully amplified in PCR (54/69 samples), although the success rate was higher for aquarium (50/54) than BB samples (4/15) (Fig. 2; Table 3). RNA yield was unrelated to PCR success (Figs 1 and 2). The four aquarium samples that failed to amplify in a PCR were all single-exhale samples that had been frozen (Fig. 1). A single PCR success was a good indicator of quality; of the samples that successfully amplified one gene and were then utilized in a second PCR targeting a different gene, 87% (46/53 samples) were successful in the second reaction.

**Table 3:** PCR performance for aquarium and BB samples by gene

Sample	PCR results (# successfully amplified/# attempted)					
	*Rpl8*	*Actb*	*Gapdh*	*IL-12p40*	*TNFα*	*Cox-2*
Aquarium, 1 exhale	17/21	11/17	7/11	4/6	4/7	5/8
Aquarium, 3 exhales	31/31	23/23	2/2	26/29	27/29	24/25
BB, 3 exhales	4/15	-	-	-	2/4	-

Among aquarium samples, the overall PCR success for housekeeping genes (96/110, 87%) was the same as the overall PCR success for immune function genes (90/104, 87%) (Table 3). A total of 37/39 (95%) of the aquarium samples tested amplified at least one transcript associated with immune function, although three samples that failed to amplify a housekeeping gene were not tested for immune function transcripts. Overall PCR success did not vary with handling regime; samples that were never frozen had similar PCR success rate (75/86, 87%) as samples that had been frozen prior to RNA extraction (111/128, 87%). However, overall PCR success was higher for aquarium samples with three exhales (133/139, 96%) than for samples consisting of a single exhale (48/70, 69%) (Table 3). PCR success for the three aquarium belugas was similar for both housekeeping genes (DL1: 43/48, 90%; DL2: 44/49, 90%; DL3: 9/13, 69%) and immune function genes (DL1: 39/46, 85%; DL2: 47/54, 87%; DL3: 4/4, 100%).

Sequencing of representative PCR products confirmed their identity. Three primer sets were found to co-amplify unintended products in addition to their targets. In 60% of the reactions, the *Rpl8* primers co-amplified a product of ~580 bp. Sequence analysis of this product demonstrated no alignments to the beluga genome. In 68% of the reactions, the *TNFα* primers co-amplified a product of ~450 bp. Sequence analysis of this product demonstrated that it was a 98% match with a 410-bp segment of an 18 s ribosomal RNA gene from a Dysteriid ciliate (Accession #: KF384514.1), a species that has been found in clinically healthy belugas (Sniezek *et al.,* 1995). In 58% of the reactions, the *IL-12p40* primers co-amplified one to three additional products between 200 and 800 bp that do not align with the beluga genome but do have high sequence similarity with the predicted sequence for bottlenose dolphin (*Tursiops truncatus*) 28S ribosomal RNA (Accession # XR_004524347.1).

## Discussion

This study has demonstrated that RNA can be extracted from beluga blow samples consisting of one to six exhales and that transcripts associated with housekeeping genes and immune function can be amplified via PCR in RNA extracted from blow. Immune function transcripts were detected in 95% of the aquarium samples tested, suggesting that longitudinal quantitative experiments such as those described in Sitt *et al.* (2016) using blood samples from cetaceans in professionally managed care would be possible using beluga blow samples. Such longitudinal monitoring would provide the physiological validation required to apply this approach more broadly. Using blow as a tissue source for quantitative gene expression studies would provide another potential diagnostic of beluga immune function and respiratory health.

The relative value of an individual sample will depend on RNA yield, which places limits on the number or type of analyses performed. RNA yield is affected by the amount of starting material, which was unknown in this study. Collecting more exhales per sample should theoretically increase the amount of starting material, but among aquarium samples, collecting three exhales did not appreciably increase yield over the collection of a single exhale. The declining mean and median RNA yield per exhale in aquarium samples suggest that the first exhale collected contributed the most to the sample. This exhale had a much higher proportion of environmental water because the whale did not submerge between exhales. Therefore, the first exhale may have had the largest quantity of cells and microorganisms compared with successive exhales. A study of the effect of the number of exhales collected and DNA yield in belugas produced similar results, with declining mean and median DNA yields per exhale as the number collected increased from one to two to four (Richard *et al.,* 2017a). Variation in blow sample volume in belugas (Richard *et al.,* 2017b) and variation in the amount of cellular debris expelled per exhale could explain the variation in RNA yield observed in this study. Yield could be improved through the use of alternative denaturants that might be better suited for mucus-rich material (Bouchard *et al.,* 2020), alternative extraction protocols such as phenol-chloroform methods, or through the application of whole transcriptome amplification protocols. Further experimentation could also be performed with the homogenization protocol used. However, the commercial kit used in this study provided a time and cost-effective protocol that allowed for PCR amplification in most samples.

The purity and integrity of the RNA sample also affects its relative value in downstream experiments. Very few of the samples had A_260_/A_280_ ratios between 1.8 and 2.0, the range considered to be optimal (Fleige and Pfaffl, 2006), yet most samples performed well in PCR regardless with beluga sequence-specific exon spanning primers. The low concentrations likely led to the erratic A_260_/A_280_ measurements of these samples. This is supported by the low, but more consistent A_260_/A_280_ values for samples with higher yields. The presence of RNA*later* mixed with environmental salt water at the start of the extraction protocol could also result in salt contamination that may interfere with nanodrop measurements, as suggested by Borowska *et al.* (2014) in a study of harbour porpoise blow samples. Nanodrop data for the RNA isolation was thus not a good predictor of PCR performance following RT. More sensitive assays of RNA purity and integrity may be more useful in predicting the downstream value of a given sample and providing further context for interpreting qRT-PCR results (Fleige and Pfaffl, 2006). Directly measuring RNA integrity would also enable comparisons between blow samples and other tissue sources used for gene expression experiments in belugas.

Handling regime did not appear to affect RNA yield. Chilling the sample for 6 h prior to freezing led to a modest increase in yield, although the sample size is too small to draw any meaningful conclusions. RNA*later* has been shown to stabilize bacterial communities in other sample types (Flores *et al.,* 2015). However, it is possible that the samples were incompletely mixed with RNA*later*, especially within clumps of mucus, allowing continued bacterial growth prior to freezing and thus larger yields. There was no evidence that chilling the samples for 6 h before freezing impacted PCR success, as all of the aquarium samples subjected to this handling regime were successful in at least one PCR. All four samples that failed to amplify any PCR products were frozen, but individual PCR failures were equally distributed among samples that had been processed fresh and those that had been frozen prior to RNA extraction. These findings suggest that samples collected, handled and stored under field conditions can be utilized for these purposes.

Both RNA yield and PCR performance were lower for BB samples compared with aquarium samples. As sample handling regime did not appear to explain this difference, the most likely explanation is the force of the exhalations, as suggested by Richard *et al.* (2017a) for similar results with DNA extraction from BB samples. Under restraint conditions, BB belugas breathe deeper, yet calmer and less forcefully than the aquarium belugas, which are trained to exhale forcefully to simulate a surfacing free-swimming beluga. Reduced force would likely reduce the amount of cellular debris expelled from the blowhole, which would reduce both DNA and RNA yields. In the study of DNA extractions from BB belugas, the effect largely disappeared when four exhales were collected (Richard *et al.,* 2017a). Several steps could be made in an effort to increase the value of samples collected from temporarily restrained belugas intended for RNA extraction, including collecting a larger portion of the blow sample by using a different collection device and collecting more exhales to improve the chance that larger pieces of cellular debris will be collected. These refinements, coupled with laboratory procedures aimed at increasing yield, would improve the applicability of this method for wild belugas.

The diversity of microorganisms in cetacean blow (Sweeney and Reddy, 2001; Raverty *et al.,* 2017, Acevedo-Whitehouse *et al.,* 2010) presents both challenges and opportunities. Microorganisms contribute an unknown amount of RNA to the total sample, which complicates the interpretation of RNA yields measured via nanodrop. In a study of pooled humpback whale blow samples, just 0.9% of the transcripts detected were of whale origin (Geoghegan *et al.,* 2018). A high proportion of microorganism to host RNA is known to inhibit PCR targeting host-specific DNA sequences (Ball *et al.,* 2007), which may explain the samples in this study with relatively higher yields that failed to amplify a PCR product. The presence of microorganisms in blow samples also means that primer pairs originally designed for use with RNA extracted from blood samples may need to be redesigned to ensure specific amplification, as indicated by the apparent co-amplification of ciliate-derived products with the *TNFα* primers used in this study. While the presence of these microorganisms can interfere with host-specific diagnostics, it simultaneously creates an opportunity to screen for various microorganisms in blow samples, as accomplished in other cetaceans (Groch *et al.,* 2020). For example, this approach could be used to assess the prevalence of SARS-CoV-2 in belugas, as they have been deemed highly susceptible to infection based on molecular markers and potential exposure through wastewater in some regions of Alaska (Mathavarajah *et al.,* 2021).

While not currently efficient enough to replace skin biopsy sampling for the study of free-swimming cetaceans, blow sampling’s less-invasive nature may lead to research opportunities that otherwise may not occur. Unmanned aerial vehicles (drones) are increasingly used to sample microbiota in blow from a variety of cetaceans, including small odontocetes (Centelleghe *et al.,* 2020). Continued development of drone technology may eventually allow for tracking and sufficiently close approaches over surfacing belugas to collect usable RNA samples. Further research on aquarium belugas can refine sampling and extraction methods and provide the necessary physiological validation for this developing diagnostic tool. For wild belugas specifically, this approach could be utilized during health assessments of temporarily restrained belugas, in populations where close-proximity boat-based sampling is feasible (Hudson *et al.,* 2021), or while they are temporarily mass-stranded, as occurs occasionally in the endangered population of Cook Inlet, Alaska (National Marine Fisheries Service, 2015). Utilizing blow samples for gene expression studies also has the distinct advantage of providing information specific to the respiratory system, which is likely to be increasingly affected by anthropogenic disturbances.

## Funding

This material is based upon work conducted at a Rhode Island National Science Foundation Established Program to Stimulate Competitive Research research facility, the Genomics and Sequencing Center, supported in part by the National Science Foundation EPSCoR Cooperative Agreement #EPS-1004057. Sampling in BB was supported by the Georgia Aquarium, the National Marine Fisheries Service and the Alaska SeaLife Center. This project was supported in part by an Undergraduate Research and Innovation grant awarded to K.S. from the University of Rhode Island and the University of Rhode Island Undergraduate Honors Program grant awarded to Hannah Kaplan.

## Data Availability

The data underlying this article will be shared on reasonable request to the corresponding author.

## References

[ref1] Acevedo-Whitehouse K , Rocha-Gosselin A, Gendron D (2010) A novel non-invasive tool for disease surveillance of free-ranging whales and its relevance to conservation programs. Anim Conserv 13: 217–225.

[ref2] Ball MC , Pither R, Manseau M, Clark J, Peterson SD, Kingston S, Morrill N, Wilson P (2007) Characterization of target nuclear DNA from faeces reduces technical issues associated with the assumptions of low-quality and quantity template. Conserv Genet 8: 577–586.

[ref3] Beineke A , Siebert U, Müller G, Baumgärtner W (2007) Increased blood interleukin-10 mRNA levels in diseased free-ranging harbor porpoises (*Phocoena phocoena*). Vet Immunol Immunopathol 115: 100–106. 17055589 10.1016/j.vetimm.2006.09.006

[ref4] Beineke A , Siebert U, Wholsein P, Baumgärtner W (2010) Immunology of whales and dolphins. Vet Immunol Immunopathol 133: 81–94. 19700205 10.1016/j.vetimm.2009.06.019

[ref5] Borowska EI , Nowak Z, van Elk C, Wahlberg M (2014) Determining genotypes from blowhole exhalation samples of harbor porpoises (*Phocoena phocoena*). Aquat Mamm 40: 407–411.

[ref6] Bouchard C , Michaels J, Brown-Harding H (2020) RNA isolation from corals and other cnidarian species using urea-LiCl as a denaturant. Anal Biochem 588: 113472. 31605694 10.1016/j.ab.2019.113472

[ref7] Buckman AH , Veldhoen N, Ellis G, Ford JKB, Helbing CC, Ross PS (2011) PCB-associated changes in mRNA expression in killer whales (*Orcinus orca*) from the NE Pacific Ocean. Environ Sci Technol 45: 10194–10202. 21985468 10.1021/es201541j

[ref8] Burek-Huntington KA , Dushane JL, Goertz CEC, Measures LN, Romero CH, Raverty SA (2015) Morbidity and mortality in stranded cook inlet beluga whales *Delphinapterus leucas*. Dis Aquat Organ 114: 45–60. 25958805 10.3354/dao02839

[ref9] Centelleghe C , Carraro L, Gonzalvo J, Rosso M, Esposti E, Gili C, Bonato M, Pedrotti D, Cardazzo B, Povinelli M et al. (2020) The use of unmanned aerial vehicles (UAVs) to sample the blow microbiome of small cetaceans. PLoS One 15: e0235537. 32614926 10.1371/journal.pone.0235537 PMC7332044

[ref10] Desforges JPW , Sonne C, Levin M, Siebert U, De Guise S, Dietz R (2016) Immunotoxic effects of environmental pollutants in marine mammals. Environ Int 86: 126–139. 26590481 10.1016/j.envint.2015.10.007

[ref11] Fair PA , Schaefer AM, Houser DS, Bossart GD, Romano TA, Champagne CD, Stott JL, Rice CD, White N, Reif JS (2017) The environment as a driver of immune and endocrine responses in dolphins (*Tursiops truncatus*). PLoS One 12: e0176202. 28467830 10.1371/journal.pone.0176202 PMC5415355

[ref12] Fleige S , Pfaffl MW (2006) RNA integrity and the effect on the real-time qRT-PCR performance. Mol Aspects Med 27: 126–139. 16469371 10.1016/j.mam.2005.12.003

[ref13] Flores R , Shi J, Yu G, Ma B, Ravel J, Goedert JJ, Sinha R (2015) Collection media and delayed freezing effects on microbial composition of human stool. Microbiome 3: 33. 26269741 10.1186/s40168-015-0092-7 PMC4534027

[ref14] Frère CH , Krzyszczyk E, Patterson EM, Hunter S, Ginsburg A, Mann J (2010) Thar she blows! A novel method for DNA collection from cetacean blow. PLoS One 5: e12299. 20811619 10.1371/journal.pone.0012299 PMC2928266

[ref15] Geoghegan JL , Pirotta V, Harvey E, Smith A, Buchmann JP, Ostrowski M, Eden JS, Harcourt R, Holmes EC (2018) Virological sampling of inaccessible wildlife with drones. Viruses 10: 300. 10.3390/v10060300 PMC6024715 29865228

[ref16] Groch KR , Blazquez DNH, Marcondes MCC, Santos J, Colosio A, Delgado JD, Catão-Dias JL (2020) Cetacean morbillivirus in humpback whales’ exhaled breath. Transbound Emerg Dis 68: 1736–1743. 33070446 10.1111/tbed.13883

[ref17] Hobbs RC , Reeves RR, Prewitt JS, Desportes G, Breton-Honeyman K, Christensen T, Citta JJ, Ferguson SH, Frost KJ, Garde E et al. (2019) Global review of the conservation status of monodontid stocks. Mar Fish Rev 81: 1–53.

[ref18] Hudson JM , Anderson WG, Marcoux M (2021) Measurement of cortisol in blow samples collected from free-swimming beluga whales (*Delphinapterus leucas*). Mar Mamm Sci 37: 888–900.

[ref19] Hunt KE , Moore MJ, Rolland RM, Kellar NM, Hall AJ, Kershaw J, Raverty SA, Davis CE, Yeates LC, Fauquier DA et al. (2013) Overcoming the challenges of studying conservation physiology cot006 in large whales: a review of available methods. Conserv Physiol 1. 10.1093/conphys/cot006. PMC4806609 27293590

[ref20] Lane SM , Smith CR, Mitchell J, Balmer BC, Barry KP, McDonald T, Mori CS, Rosel PE, Rowles TK, Speakman TR et al. (2015) Reproductive outcome and survival of common bottlenose dolphins sampled in Barataria Bay, Louisiana, USA, following the Deepwater Horizon oil spill. Proc Biol Sci 282: 20151944. 26538595 10.1098/rspb.2015.1944 PMC4650159

[ref21] Mathavarajah S , Stoddart AK, Gagnon GA, Dellaire G (2021) Pandemic danger to the deep: the risk of marine mammals contracting SARS-CoV-2 from wastewater. Sci Total Environ 760: 143346. 33160659 10.1016/j.scitotenv.2020.143346 PMC7598747

[ref22] National Marine Fisheries Service (2015) Draft Recovery Plan for the Cook Inlet Beluga Whale (*Delphinapterus leucas*). National Marine Fisheries Service, Alaska Regional Office, Protected Resources Division, Juneau, AK.

[ref23] Noël M , Loseto LL, Helbing CC, Veldhoen N, Dangerfield NJ, Ross PS (2014) PCBs are associated with altered gene transcript profiles in Arctic beluga whales (*Delphinapterus leucas*). Environ Sci Technol 48: 2942–2951. 24490950 10.1021/es403217r

[ref24] Norman SA , Goertz CEC, Burek KA, Quakenbush LT, Cornick LA, Romano TA, Spoon T, Miller W, Beckett LA, Hobbs RC (2012) Seasonal hematology and serum chemistry of wild beluga whales (*Delphinapterus leucas*) in Bristol Bay, Alaska, USA. J Wildl Dis 48: 21–32. 22247370 10.7589/0090-3558-48.1.21

[ref25] Pasamontes A , Aksenov A, Schivo M, Rowles T, Smith CR, Schwacke LH, Wells RS, Yeates L, Venn-Watson S, Davis CE (2017) Noninvasive respiratory metabolite analysis associated with clinical disease in cetaceans: a Deepwater Horizon oil spill study. Environ Sci Technol 51: 5737–5746. 28406294 10.1021/acs.est.6b06482

[ref26] R Core Team . (2020) R: A language and environment for statistical computing. R Foundation for Statistical Computing, Vienna, Austria. https://www.R-project.org/.

[ref27] Raverty SA , Rhodes LD, Zabek E, Eshghi A, Cameron CE, Hanson MB, Schroeder JP (2017) Respiratory microbiome of endangered southern resident killer whales and microbiota of surrounding sea surface microlayer in the eastern North Pacific. Sci Rep 7: 394. 28341851 10.1038/s41598-017-00457-5 PMC5428453

[ref28] Richard JT , Robeck TR, Osborn SD, Naples L, McDermott A, LaForge R, Romano TA, Sartini BL (2017b) Testosterone and progesterone concentrations in blow samples are biologically relevant in belugas (*Delphinapterus leucas*). Gen Comp Endocr 246: 183–193. 27989435 10.1016/j.ygcen.2016.12.006

[ref29] Richard JT , Schultz K, Goertz C, Hobbs R, Romano TA, Sartini BL (2017a) Assessing the quantity and downstream performance of DNA isolated from beluga (*Delphinapterus leucas*) blow samples. Aquat Mamm 43: 398–408.

[ref30] Sitt T , Bowen L, Blanchard MT, Smith BR, Gershwin LJ, Byrne BA, Stott JL (2008) Quantitation of leukocyte gene expression in cetaceans. Dev Comp Immunol 32: 1253–1259. 18572242 10.1016/j.dci.2008.05.001

[ref31] Sitt T , Bowen L, Lee C, Blanchard MT, McBain J, Dold C, Stott JL (2016) Longitudinal evaluation of leukocyte transcripts in killer whales (*Orcinus orca*). Vet Immunol Immunopathol 175: 7–15. 27269787 10.1016/j.vetimm.2016.04.011

[ref32] Sniezek JH , Coats DW, Small EB (1995) *Kyaroikeus cetarius* N. G., N. Sp.: a parasitic ciliate from the respiratory tract of odontocete cetacea. J Eukaryot Microbiol 42: 260–268.

[ref33] Sweeney JC and Reddy ML. (2001) Cetacean cytology. In Dierauf LA and Gulland FMD, eds. CRC Handbook of Marine Mammal Medicine: Health, Disease, and Rehabilitation, 2nd Edition. New York: CRC Press, 437 pp., 10.1201/9781420041637.ch20.

[ref34] Thompson LA , Spoon TR, Goertz CE, Hobbs RC, Romano TA (2014) Blow collection as a non-invasive method for measuring cortisol in the beluga (*Delphinapterus leucas*). PLoS One 9: e114062. 25464121 10.1371/journal.pone.0114062 PMC4252093

[ref35] Unal E , Goertz CEC, Hobbs RC, Suydam R, Romano T (2018) Investigation of molecular biomarkers as potential indicators of health in wild belugas (*Delphinapterus leucas*). Mar Biol 165: 182. 10.1007/s00227-018-3439-3.

[ref36] Venn-Watson S , Colegrove KM, Litz J, Kinsel M, Terio K, Saliki J, Fire S, Carmichael R, Chevis C, Hatchett W et al. (2015) Adrenal gland and lung lesions in Gulf of Mexico common bottlenose dolphins (*Tursiops truncatus*) found dead following the Deepwater Horizon oil spill. PLoS One 10: e0126538. 25992681 10.1371/journal.pone.0126538 PMC4439104

